# Could immunotherapy and regulatory T cells be used therapeutically to slow the progression of Alzheimer’s disease?

**DOI:** 10.1093/braincomms/fcaf092

**Published:** 2025-02-25

**Authors:** Victoria Abbott, Benjamin E Housden, Annwyne Houldsworth

**Affiliations:** Neuroscience, Clinical and Biomedical Sciences, University of Exeter Medical School, Exeter EX2 4TH, UK; Clinical and Biomedical Sciences, University of Exeter Medical School, Exeter EX2 4TH, UK; Living Systems Institute, University of Exeter, Exeter EX4 4QD, UK; Neuroscience, Clinical and Biomedical Sciences, University of Exeter Medical School, Exeter EX2 4TH, UK; Clinical and Biomedical Sciences, University of Exeter Medical School, Exeter EX2 4TH, UK

**Keywords:** regulatory T cells, Alzheimer’s disease, oxidative stress, neuroinflammation, immunotherapy

## Abstract

Alzheimer’s disease and other cognitive impairments are a growing problem in the healthcare world with the ageing population. There are currently no effective treatments available; however, it has been suggested that targeting neuroinflammation may be a successful approach in slowing the progression of neurodegeneration. Reducing the destructive hyperinflammatory pathology to maintain homeostasis in neural tissue is a promising option to consider. This review explores the mechanisms behind neuroinflammation and the effectiveness of immunotherapy in slowing the progression of cognitive decline in patients with Alzheimer’s disease. The key components of neuroinflammation in Alzheimer’s disease researched are microglia, astrocytes, cytokines and CD8+ effector T cells. The role of oxidative stress on modulating regulatory T cells and some of the limitations of regulatory T cell–based therapies are also explored. Increasing regulatory T cells can decrease activation of microglia, proinflammatory cytokines and astrocytes; however, it can also increase levels of inflammatory cytokines. There is a complex network of regulatory T cell interactions that reduce Alzheimer’s disease pathology, which is not fully understood. Exploring the current literature, further research into the use of immunotherapy in Alzheimer’s disease is vital to determine the potential of these techniques; however, there is sufficient evidence to suggest that increasing regulatory T cells count does prevent Alzheimer’s disease symptoms and pathology in patients with Alzheimer’s disease. Some exciting innovative therapies are muted to explore in the future. The function of regulatory T cells in the presence of reactive oxygen species and oxidative stress should be investigated further in patients with neurogenerative disorders to ascertain if combination therapies could reduce oxidative stress while also enhancing regulatory T cells function. Could methods of immunotherapy infuse exogenous functional Tregs or enhance the immune environment in favour of endogenous regulatory T cells differentiation, thus reducing neuroinflammation in neurodegenerative pathology, inhibiting the progression of Alzheimer’s disease?

## Teaser

Abbott *et al.* describes Alzheimer’s disease pathology associated with neuroinflammation. Tregs are neuroprotective CD4+ T-cells that maintain central nervous system homeostasis by reducing inflammation and inhibiting T cell proliferation and cytokine production. They also reduce microglia-mediated inflammation, inhibit neurotoxicity, neuroinflammation and the progression of neurofibrillary tangles and Aβ production.

## Graphical Abstract

**Graphical Abstract:**
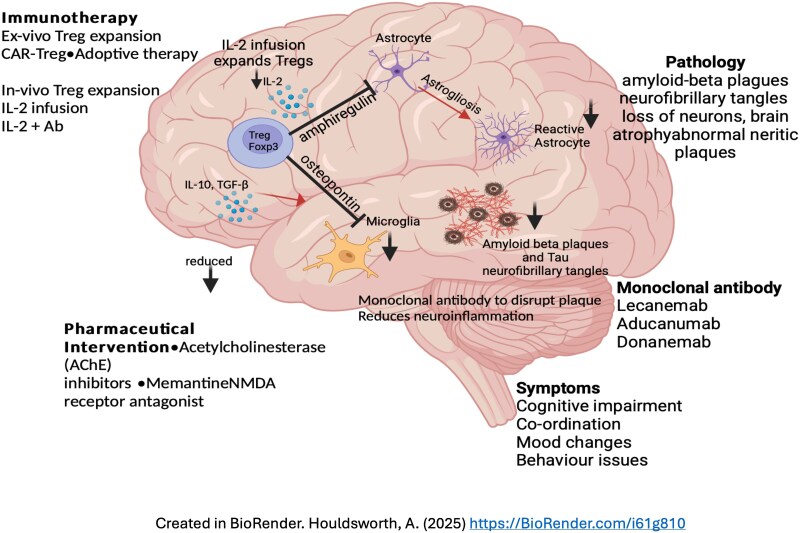

## Alzheimer’s disease symptoms and prevalence

Alzheimer's disease is a common neurodegenerative disease characterized by the progressive loss of memory, cognitive function and independence in daily tasks, where 1 in 14 of 65 years old and above are affected as late-onset Alzheimer's disease.^1‐5^ Patients presenting at a younger age, <65 years are referred to as early-onset Alzheimer's disease.^6^ Treatment for Alzheimer's disease is extremely costly worldwide estimated at £26.3 billion in the UK.^4^ Strategies to treat both cognitive decline (CD), including Alzheimer's disease and mild cognitive impairment (MCI) are important areas to address.^3,7‐10^ Criteria for pathological diagnosis of Alzheimer's disease include the presence of extracellular amyloid-beta (Aβ) plaques and intraneuronal neurofibrillary tangles (NFT) made of phosphorylated tau protein. Both abnormal accumulations of protein lead to a disruption in cell function, neuroinflammation and oxidative stress, all which have been linked to neuronal cell death.^11,12^

## Current treatments, prevention and risk factors

Research has identified multiple key risk factors for developing Alzheimer's disease. These include age, genetics and family history. According to most of the literature produced on the subject, age is the most significant risk factor for CD and Alzheimer's disease. Reports suggest that the estimated 19% prevalence of Alzheimer's disease in people ages 74–85 increases to between 30 and 50% in populations over the age of 85.^13‐16^ Therefore, preventions that aim to increase the age at onset (AAO) have a clear benefit as, especially with the ageing population, the burden of care on families and the NHS will become unsustainable.

In order to understand the pathogenesis of Alzheimer's disease, neuroinflammatory immunology and oxidative stress, the use of genome-wide association studies (GWAS) has allowed researchers to identify genetics as risk factors for Alzheimer's disease may give further clues as to the pathogenesis of the disease, especially where some polymorphisms cause reduced or dysfunctional function. There is sufficient evidence to show that multiple genetic variants, including apolipoprotein E-ε4 (*APOE*), amyloid precursor protein (*APP*), presenilin 1 (*PSEN1*) and presenilin 2 (*PSEN2*), increase an individual’s risk for AD.^17,18^ However, given that neither genetics or age are modifiable factors, research must focus on understanding and targeting modifiable factors to be effective in the search for effective preventions and therapeutics. *APOE* is overexpressed following oxidative stress leading to a greater degree of oxidative insults in the brain.^19^

Currently, there is no cure for CD or Alzheimer's disease, although there are multiple treatments offered by the NHS aimed to reduce symptoms but not reverse or stop them.^20,21^ These treatments include acetylcholinesterase (AChE) inhibitors (which increase acetylcholine levels in the brain and therefore increase nerve transmission^22^) and memantine (NMDA receptor antagonist^23^), as well as cognitive therapies.^20^ Several studies have shown the limited efficacy of these current treatments, although all three techniques are considered to delay the progression of CD and AD.^22‐26^ Current research focuses on the treatment of Alzheimer's disease through immunotherapy with multiple drugs showing promising results in delaying on the onset of CD and progression of Alzheimer's disease. In 2023, the use of monoclonal antibodies that target Aβ, Lecanemab and aducanumab (Aduhelm) were approved by the by the Food and Drug dministration (FDA) to treat AD.^27^ The drug is an intravenous infusion therapy using monoclonal antibodies to target Aβ plagues,^27,28^ similarly to a drug, Aducanumab, which was approved in 2021. Few studies have evaluated the effectiveness of Lecanemab on later stage Alzheimer's disease, thus further studies are required to determine the impact of the drug on these patients.^29^ Aducanumab was discontinued in 2024, reportedly due to the manufacturing costs,^30,31^ highlighting the major issue with monoclonal antibody therapies not being assessable. Many studies also debated the drug’s clinical efficacy and safety,^32‐34^ which lead to the drug not being approved for use in Europe and the UK.^30^ Nevertheless, Aducanumab and Lecanemab opened the door into this new era of Alzheimer's disease drug research focusing on immunotherapy to combat Alzheimer's disease symptoms.^29^ Disease-modifying drugs, such as these, have the potential to decrease the number of MCI cases developing into dementia and increase the life expectancy and quality of life for patients with Alzheimer’s disease.^10^ Overall, there is a growing need for effective Alzheimer’s disease treatments without side effects, such as serious symptoms experienced in 1–2% of patients treated with these drugs who report headache, seizures, delirium, impaired speech, problems with vision and microbleeds in the brain. Recently, Donanemab was approved by the Food and Drug Administration (FDA) to treat AD.^35^ Although Donanemab has been approved by the FDA in the United States and is available for patients, it is not able to be prescribed in the UK on the NHS, while being available to private health patients. Donanemab was trialled in patients who were experiencing early symptoms of Alzheimer’s disease with tau and amyloid pathology and was shown to slow down clinical progression at 76 weeks.^35^

## Search strategy

### Regulatory T cells and the immune role in Alzheimer’s disease

**Figure 1:**
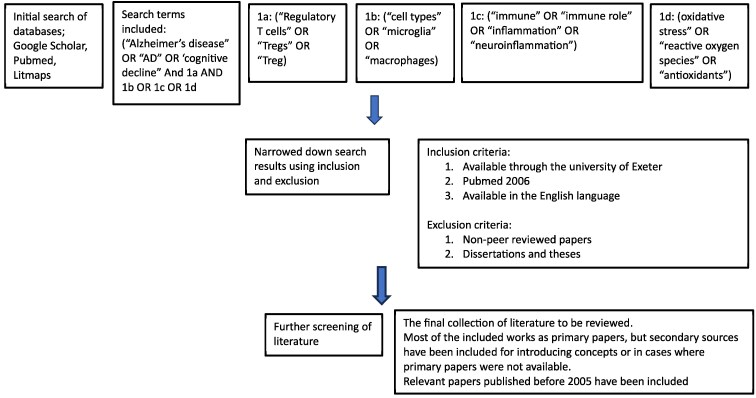
**Search strategy for discussion.** The databases listed above were searched using the terms described above. Each sections search results were then defined further, ending with a final screening for relevance.

In recent years, there has been a significant shift of focus in the literature onto the importance of the immune system and its role in Alzheimer’s disease (Fig. 1).^21,36,37^ A key example being the use of monoclonal antibodies to treat Aβ plagues.^27‐30,32‐34^ Accumulating research suggests that there is potential for therapeutics for Alzheimer’s disease using immunotherapy to target neuroinflammation, a key aspect of Alzheimer’s disease.^21,36‐38^ One of these methods of modifying the immune response is the use of *ex vivo* expanded regulatory T cell (Treg) infusions.^39^ Tregs are a subset of neuroprotective CD4+ T cells, which are identified by the expression of forkhead box protein P3 (*Foxp3*).^40^ The main role of Tregs is to maintain homeostasis within the central nervous system (CNS), by inhibiting T cell proliferation and cytokine production.^41‐43^ Tregs also have been shown to modulate adaptive immune reactions and reduce microglia and macrophage-mediated inflammation.^41^ Microglia have been suggested to accelerate neurotoxicity, neuroinflammation and the progression of NFT and Aβ pathogenesis in Alzheimer’s disease.^37,39,43^ Furthermore, studies have found that this cell population is dysfunctional in multiple neurodegenerative diseases.^39,44^ Thus, increasing Treg count may result in slower pathogenesis,^39,41^ demonstrating that Tregs have clear potential as a therapeutic target in Alzheimer’s disease. Also, Tregs play a crucial role in autoimmunity, but there is limited research on the effects of disrupting this balance and the risk of neuronal damage.^42,45^ Recently, research has focused on the different cell types affected by Tregs,^39,41‐43,46^ the effect of oxidative stress^47^ and the debated therapeutic potential of the use of Treg infusions.^41,46,48,49^ Whilst theoretically promising, the complex web of mechanisms involving Tregs in Alzheimer’s disease is not entirely understood.

Healthy brains of mice and humans have populations of brain resident CD69 Tregs, and these have been shown to expand rapidly in several neuroinflammatory conditions. Brain-resident Tregs are proposed to control astrogliosis and restrain inflammatory responses and polarize microglia into neuroprotective states.^50^

In this review, the term ‘natural Tregs’ refers to the Tregs in the body, which have not been manipulated, expanded artificially or transplanted by researchers. These cells are the Tregs present in the body that naturally occur, with varying levels based on a multitude of factors. The term ‘Treg infusions’ will be used in this review to refer to techniques that increase the frequency of Tregs within the body, including adoptive cell transfer, which uses Tregs from a donor,^51^ as well as the use of *ex vivo* expanded Tregs, which involves taking the cells out of the body and proliferating them, before returning them back to the body.^39,41^

### Research questions and aims

Several research questions are posed in this review, including, what are the mechanisms of action of Tregs on cell types in the brain; does an increase in the Treg count produce neuroprotection against Alzheimer’s disease; what side effects are there in Treg infusion and how can these findings be translated into effective therapies?

The review aims to assess the therapeutic potential of *ex vivo* expanded Treg infusions by exploring the key cellular mechanisms underlying Tregs. This research will help develop understanding of the role of Tregs in Alzheimer’s disease, investigate any underlying limitations to the proposed therapy and suggest future research to aid in the development of novel treatments for Alzheimer’s disease.

## Evidence for increasing Treg levels in Alzheimer’s disease

### Treg levels are reduced in Alzheimer’s disease

A variety of studies have established that Tregs play a critical role in homeostasis of the immune system.^42,48,52^ Given that the immune role in Alzheimer’s disease studies is an increasingly significant area of interest,^21,36,38^ Alzheimer’s disease Treg studies have become a major focus in the field.^43,46,47,53^ There is some inconsistency within the literature surrounding the levels and functionality of Tregs in Alzheimer’s disease, but most studies report a depletion of Treg count and function.^54^ For example, a 2010 study found that percentages of Treg in blood are decreased in patients with Alzheimer’s disease compared with healthy controls, with the lowest percentages of all subtypes of Tregs in patients with more severe AD.^55^ Interestingly, a more recent study found that there was no significant difference between Treg count in patients with Alzheimer’s diseasecompared to MCI controls^53^; however, the lack of healthy controls in this study may account for the inconclusive data. The reported reduction in Tregs in patients with Alzheimer’s disease implies that there may be a benefit in increasing levels to match healthy control pairs.

### Treg function is reduced in Alzheimer’s disease

In addition to the reduced frequencies of Tregs reported in patients with Alzheimer’s disease, there is evidence to suggest that the Tregs present in Alzheimer’s disease have reduced suppressive function and different immunophenotypes to healthy patient Tregs,^39^ contrasting with healthy brain Tregs, as described earlier.^50^ This study also found that there are significant differences in immunophenotypes present in patients with Alzheimer’s disease, with reduced *CD25* surface expression on Alzheimer’s disease Tregs.^39^ The CD25 marker is reportedly crucial for the survival and expansion of Tregs,^54,56^ supporting the theory that reduced frequencies of Tregs are present in Alzheimer’s disease. Conversely, to the previous study, Rosenkranz *et al*.^57^ found an increase in suppressive activity in patients with Alzheimer’s disease in comparison to young healthy controls, but no difference when compared to age-matched controls. In addition, another study found no significant difference in the percentages of CD25− Tregs between healthy controls and patients with Alzheimer’s disease, but they did find lower percentages of this subtype of Treg in individuals with MCI. This study was unable to explore the functions of Tregs due to insufficient numbers of cells from donors, which may have affected other data in their investigation.^58^ Despite the inconsistencies in findings in these studies, most researchers appear to have accepted the theory that Tregs in patients with Alzheimer’s disease have reduced function^39,41,59^ due to numbers, although the mechanism behind this and whether there are differences in immunophenotypes remains unclear.

### Increasing Tregs reduces the symptoms of Alzheimer’s disease

It is important to discuss the effect of Tregs on the symptoms of Alzheimer’s disease. Whilst there is evidence that Treg infusions reduce and prevent Alzheimer’s disease pathology,^39,46^ this reduction needs to translate into the symptoms of Alzheimer’s disease to be an effective treatment. The literature suggests that Tregs play a vital role in modulating disease progression in Alzheimer’s disease mice models. Baek *et al*.^59^ showed that lower percentages of Tregs increase the rate of CD and increase Aβ plagues. Thus, increasing Treg levels in blood could attenuate the progression of Aβ plagues. Supporting this, other studies demonstrated that amplification of Tregs restored cognitive function and delayed the onset of CD in mice models expressing chimeric mouse/human amyloid precursor and a mutant human presenilin 1 protein (APP/PS1).^60‐63^ Together, these studies suggest that Tregs are essential in learning and memory, which are key aspects of Alzheimer’s disease. The data also suggest that the immunosuppressive role of Tregs may be the key in the prevention of Alzheimer’s disease pathology. Although this is a new field of research in Alzheimer’s disease and currently, there is sparse data about how these findings translate into human patients, some recent findings demonstrate a clear potential in further research to determine the extent to which Treg therapies can reduce/prevent symptoms of Alzheimer’s disease.

The main focus of this review has explored *ex vivo* Treg expansion, it is also important to consider *in vivo* Treg expansion as a promising area of research and therapeutic approach, involving the adoptive transfer of these immune cells. Tregs express an abundance of interleukin-2 receptors and congenital deficiency or dysfunction of this receptor reduces the differentiation of Treg cell lineage, thus has been shown to be essential for the development of Tregs.^64^ In a recent phase 1 trial of amyotrophic lateral sclerosis (ALS), patients receiving interleukin-2 and CTLA-4Ig, biomarkers of OS were observed as well as indicators of neurodegeneration and neuroinflammation.^65,66^ Recombinant human IL-2 (Aldesleukin) has been administered in a phase 2a trial for patients with ALS, in a randomized, double-blind, placebo-controlled trial and found to expand the Treg population, reducing plasma myeloid activating cytokines, while suppressing peripheral proinflammatory monocytes.^66,67^ This trial has served as a proof-of-concept trial as to the impact of *in vivo* Treg expansion on patients with Alzheimer’s disease. It has yet to be determined if this type of therapy for *in vivo* Treg expansion will modify neuroinflammation in patients with AD.^67^

## Tregs can be modulated by various factors

### Oxidative stress promotes instability of Tregs

Oxidative stress describes an imbalance between free radicals and antioxidants. Free radicals are highly reactive molecules, due to an uneven number of electrons, which can bind to other molecules to result in damage to DNA, proteins and tissue when in excess.^68‐71^ Reactive oxygen species (ROS) are a subset of free radicals containing oxygen that can induce inflammation,^72^ which is shown to be a key player in Alzheimer’s disease pathology.^3,37,73^ Oxidative stress has often been associated with CD and AD,^74,75^ and there is evidence to suggest that Tregs may play a role in this mechanism. As previously mentioned, data suggest that patients with Alzheimer’s disease may have an increased percentage of CD25− Tregs,^39^ and CD25 knockout Tregs have been shown to increase oxidative stress and apoptosis.^56^ Furthermore, there is evidence that oxidative stress promotes instability of Tregs.^47^ Together with the previous study, this demonstrates there is a clear link between ROS and Tregs in the progression of Alzheimer’s disease. Understanding this link may be useful to determine the therapeutic potential of Tregs. It is possible that the higher levels of oxidative stress found in patients with Alzheimer’s disease may decrease the efficacy of Treg infusions in comparison to the use of similar methods to treat other diseases, but there is yet to be any research investigating this. There is also evidence that microglia may be activated by excessive ROS, leading to further neuroinflammation.^68^ The studies imply that there is a benefit to increasing Treg levels in Alzheimer’s disease, as the association between oxidative stress and Alzheimer’s disease may be partially due to the instability of Tregs caused by ROS.

Furthermore, oxidative damage leads to an increase in IL-6 production, which has been shown to inhibit Foxp3 expression in Treg differentiation,^76^ suggesting that oxidative stress is the cause of a decrease of Treg levels in patients with Alzheimer’s disease. Since this inhibition of expression takes place in the differentiation of the cells, oxidative stress may not affect infused Tregs; however, more research is needed to investigate this.

### Superoxide dismutases can modulate oxidative stress

Superoxide dismutase (SOD) enzymes catalyze the dismutation of superoxide anions (a type of ROS) and play a central role in defence against oxidative stress. They are typically associated with metals, such as copper, zinc, iron or manganese.^68,77^ In Alzheimer’s disease, mutations in the gene encoding for SOD2 have been identified, resulting in a loss of antioxidant activity.^68^ Interestingly, SOD2 is dependent on manganese, which has also been found to be significantly reduced in AD.^78^ There is evidence to suggest that SOD2 is upregulated in patients with Alzheimer’s disease, although this may be a compensatory mechanism due to the increased oxidative stress.^78^ Studies have shown that reducing SOD2 activity in Alzheimer’s disease models increases disease-like pathology,^70^ demonstrating clearly that oxidative stress and SOD enzymes play an important role in Alzheimer’s disease pathology. Using this, Treg levels could potentially be increased naturally by increasing the activity of SOD. Given the dysfunctional role of SOD enzymes in Alzheimer’s disease, it is possible that an infusion of Tregs would have a limited effect as they become unstable from ROS, and it may be necessary to measure the antioxidant status of patients with Alzheimer’s disease before administering these novel treatments. Further research is necessary to better understand the mechanisms of oxidative stress and Tregs.

### Antioxidants can modulate oxidative stress

Antioxidant-rich food is well known to have many health benefits. Antioxidants are natural polyhydroxylated phenolic compounds with low molecular weights and can eliminate ROS. Antioxidants reduce oxidative stress and therefore can be used to prevent AD.^71^ Many of these foods have been demonstrated to alter Aβ aggregation, improve neuronal physiology and enhance cognitive abilities.^79‐81^ This demonstrates again that ROS plays an important role in Alzheimer’s disease pathology. Whilst there are issues with antioxidants crossing the blood–brain barrier,^81^ advancements in therapies for Alzheimer’s disease could produce a drug that uses antioxidants alongside Treg-based therapies to target both aspects of the disease. Potentially, creating a drug with a lower dosage of Tregs, requiring fewer intravenous infusions and reduced side effects, may provide a more assessable treatment. Without elucidating the mechanisms of oxidative stress in Alzheimer’s disease, it is possible that new Tregs would not differentiate, and Treg infusions will not have long-term effects.

## Tregs mechanism of action in Alzheimer’s disease

### Tregs modulate neuroinflammation

**Figure 2:**
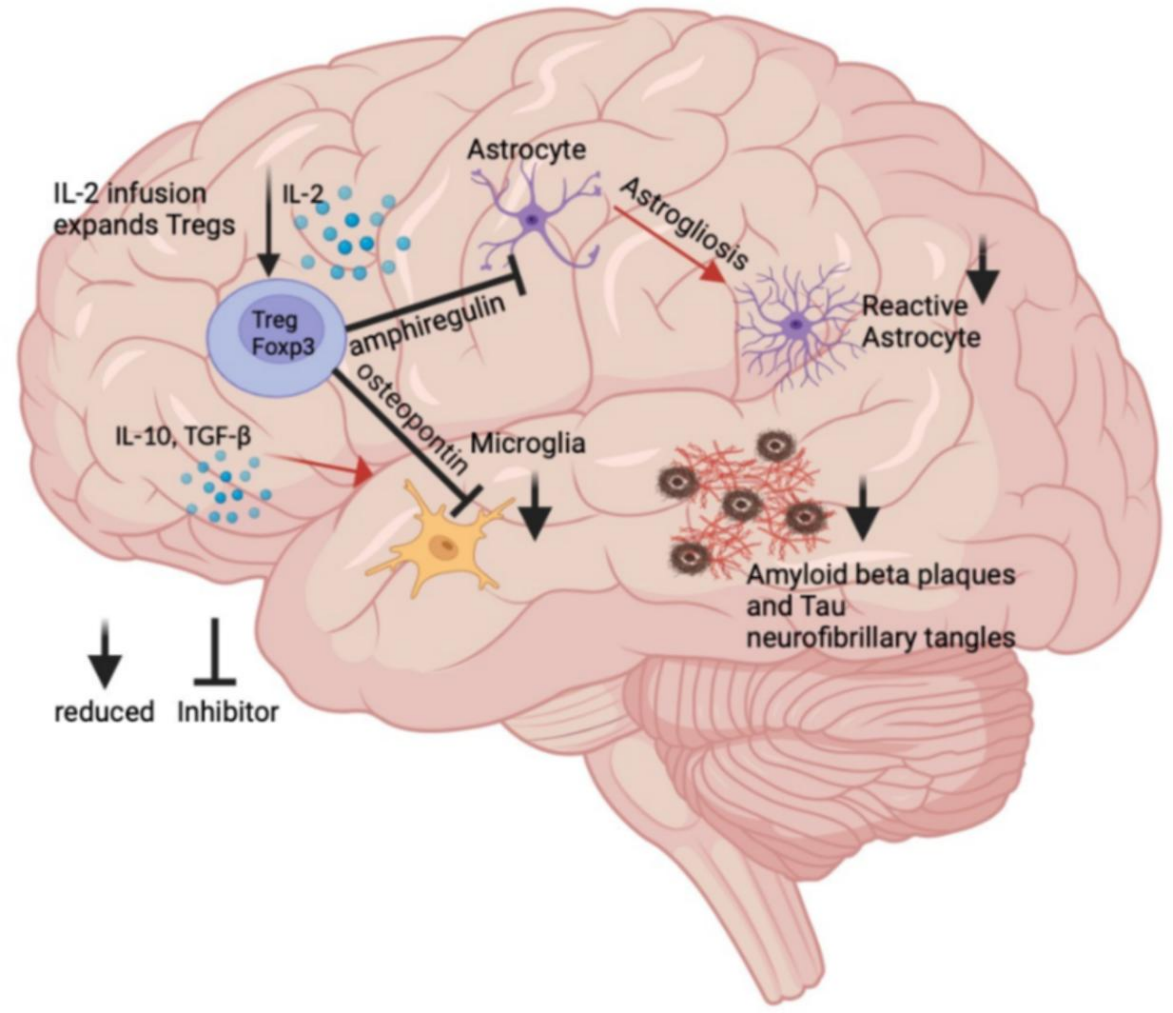
**The mechanisms underpinning the effect of increased regulatory T cells on neuroinflammation.** Regulatory T cells (Tregs) modulate many pathways in Alzheimer's disease to reduce inflammation. Tregs modulate astrogliosis to prevent reactive astrocytes.^85^ They also inhibit microgliosis.^58^ Both functions lead to reduced amyloid-beta plagues and neurofibrillary tangles of tau protein.^61,62^ Furthermore, Tregs produce many anti-inflammatory cytokines such as interleukin-10 (IL-10) and transforming growth factor-beta TGF-β.^86^ Image adapted from Jaber^87^ and information from Le Page *et al*.,^58^ Chay,*et al*.,^85^ Park *et al*.^86^ and Chakrabarty *et al*.^88^ Created in BioRender. Houldsworth, A. (2025) https://BioRender.com/i62v491.^89^

Extensive research has shown that Tregs play an important role in modulating neuroinflammation in Alzheimer’s disease.^21,37,38,41^ Neuroinflammation has been suggested as an essential therapeutic target due to the role of neuroinflammation in aggravating Aβ pathology and Alzheimer’s disease development.^82,83^ There is also evidence to suggest that ongoing inflammation aids the phosphorylation of tau.^73,84^ There are various known mechanisms underpinning this neuroinflammation that will be discussed in the next section, with focus on the interactions Tregs have with different cell types (Fig. 2) and how these affected cells interact with Aβ and tau pathologies.

### Tregs modulate microglial activation

Microglia act as the first defence in the brain by phagocytosing dead cells and cell debris and in Alzheimer’s disease, microglia respond to Aβ plague. Microglia also phagocytose misfolded protein, such as both soluble and fibrillary forms of Aβ.^90,91^ These cells can also trigger both pro-inflammatory and anti-inflammatory cytokines into action. Whilst the preliminary activation of microglia is vital for containing and preventing Aβ plaque, sustained activation leads microglia to skew towards defective subtype, which can cause long-term damage to the CNS.^91‐93^ These microglia increase Aβ plagues and tau proliferation, which then activate more microglia, creating a self-proliferating pathology.^94,95^ The suppressive function of Tregs can regulate the inflammatory microglial phenotype.^51^ This has been demonstrated using *ex vivo* expansion and adoptive transfer studies, showing the increased numbers and function of Tregs can reduce the levels of reactive microglia, as well as altering the inflammatory phenotype.^39,51,59^

Further suggesting the significance of microglia in Alzheimer’s disease pathology, GWAS have identified the APOE E4 gene variant (which is found in a gene that are expressed by microglia) to be associated with AD.^91,96^ A 2022 study also found that the function of microglial Aβ clearance is compromised in Alzheimer’s disease, although they found this can be improved by activation of PIEZO1 channels.^97^ The complex mechanisms in which microglial function is reduced in Alzheimer’s disease is not yet fully understood, further studies exploring this may provide insight into finding new therapeutic targets for understanding and treating Alzheimer’s disease.

Although studies show Treg-induced reduction in microglia to have a positive effect in reducing and preventing Aβ plague,^39,41,51,59^ there remains a question about the discriminatory abilities and localization of Tregs.^98^ The balance of microglia is important to maintain normal functioning as they play a crucial role in modulating synaptic plasticity through the expression and release of neurotrophic factors, such as brain-derived neurotrophic factor (BDNF). BDNF is released via the microglial phosphatidylinositol 3-kinase/BDNF signalling pathway and is essential for neuronal growth, learning and memory.^99‐102^ It has been shown that decreased levels of BDNF are associated with Alzheimer’s disease, suggesting that BDNF may be useful in the prevention of AD.^99^ Whilst this review is limited to the role of BDNF as a microglial-derived neurotrophic factor as an example, it is important to recognize that there are various other microglial-derived neurotrophic factors that are affected by microglial count and may play a role in Alzheimer’s disease. Therefore, the control of Tregs function, migration and survival is essential in reducing microglial-meditated Alzheimer’s disease pathology and further research into controlling and understanding the balance of Tregs and microglia would prove beneficial in the steps towards a safe and effective Treg drug for Alzheimer’s disease. Studies trialling *ex vivo* expanded Tregs on animal models fail to mention the importance of exploring this balance to develop an effective drug. In the future, it would be interesting to see studies focused on the potential negative impact of excessive Tregs on microglia in Alzheimer’s disease brains to better understand the consequences of over-dosing this new drug type.

Some abnormal signalling has been reported across a network of microglial genes in their expression of mRNA transcript alterations as a shared genetic risk in neurodegenerative disorders. These aberrant gene expressions affect physical interactions between CCRX4 (CD152), a receptor on Tregs, and microglial receptors. This changed signalling may contribute to neurodegeneration.^103^ However, *ex vivo* expanded adoptive transfer Tregs modulate the activation and polarization of microglia in TMT-induced neurodegeneration animal models, and this may be a mechanism that could shift the activation of microglia from inflammatory clustered forms to quiescent conformations, thus reducing the formation of dystrophic neurites.^86^

### Tregs activate cytokines

Another way that Tregs can act to modulate neuroinflammation is by producing immunosuppressive cytokines, such as interleukin 10 (IL-10), interleukin 35 (IL-35) and transforming growth factor beta.^104^ Cytokines play an important role in regulating immune responses and therefore can modulate neuroinflammation in Alzheimer’s disease. For example, IL-10 suppresses inflammation by inhibiting the secretion of IL-1β, IL-6 and tumour necrosis factor alpha by microglia.^105^ A study by Le Page *et al*.^58^ found IL-10 levels to be higher in both Alzheimer’s disease and MCI patients in comparison to healthy controls. However, the effects of IL-10 on Alzheimer’s disease pathology are still debated, with some studies showing excessive IL-10 in Alzheimer’s disease models to worsen CD and Aβ plague,^105^ and others showing increased levels of IL-10 to improve spatial learning in APP/PS1 mice.^106^ Despite this, transforming growth factor beta has been shown to promote microglial and macrophage-mediated Aβ clearance^107^ demonstrating that cytokines play a significant role in Alzheimer’s disease. Further investigation of cytokine interactions in Alzheimer’s disease is recommended to better understand the significance of these within the disease.

There is sufficient evidence to suggest that interleukin-2 (IL-2) improves amyloid pathology and memory in Alzheimer’s disease mice.^108^ Interestingly, IL-2 also boosts the suppressive function of Tregs not only suggesting that Tregs also may a role in improving Alzheimer’s disease hallmarks but also highlighting IL-2 as a potential target for Treg enhancement and Alzheimer’s disease treatment.^108,109^ IL-2 is necessary for *Foxp3* expression and signalling to maintain survival of Tregs,^110,111^ thus vital for development and stability. This is demonstrated further by studies using IL-2 treatments to amplify Tregs, which have been shown in restore cognitive functions in APP/PS1 mice.^60^ However, there is a low presence of IL-2 in Alzheimer’s disease brains and Tregs are poor at producing IL-20.^112,113^ Taken together, this suggests that for Treg infusions to have long-term effects in Alzheimer’s disease, levels of IL-2 may also need to be increased within the CNS.

Both pro-inflammatory and anti-inflammatory cytokines are elevated in the plasma of patients with Alzheimer’s disease, reflecting the changes in the immune system.^58,114^ It is unclear whether this is a consequence or causing factor of Alzheimer’s disease, highlighting the need for further research into the dynamics of the disease. Whilst the mechanisms and impact of some of the Treg-induced cytokines on Alzheimer’s disease may be partially unclear, the fact that Tregs have been shown to produce immunosuppressive cytokines remains and is therefore relevant to study further when exploring the therapeutic potential of Tregs.

### Tregs modulate astrocytes

Astrocytes make up a significant part of the CNS; however, for decades, they were believed to play just a supporting role in the brain and were largely overlooked.^115^ Astrocytes have many structural, metabolic and neuroprotective functions, including the modulation of synapses.^116,117^ As part of the immune response, astrocytes undergo reactive astrogliosis, a process which includes increased gene expression of structural proteins and proliferation. In Alzheimer’s disease, reactive astrocytes build up around Aβ plagues, have chronic activation and dysfunctional signalling.^118,119^ Studies have shown that excessive Aβ upregulates astrogliosis,^85^ thus reactive astrocytes play a key role in neuroinflammation in Alzheimer’s disease. Similarly to microglia, Tregs can modulate astrocytes to prevent neuroinflammation.^120^ Studies have also found that a depletion of Tregs increases the activation of astrocytes,^121^ supporting the benefit of increasing Tregs as a method to prevent neuroinflammation in Alzheimer’s disease.

There is evidence that astrocytes can protect the CNS from oxidative injury. However, other reports suggest astrocytes may be a source of ROS and in turn, excessive ROS may lead to may reactive astrocytes,^69,122^ suggesting they play a dual role. Astrocytes can also phagocytose Aβ and secrete Aβ-degrading proteases, such as APOE.^123^ Whilst astrocytes play an important role in maintaining a healthy CNS, the balance of reactive astrocytes is critical in preventing neuroinflammation. Exploring the mechanisms that Tregs modulate astrocytes is critical for safe application of Treg-based therapies to prevent over-modulation of vital astrocytes.

### Tregs modulate CD8+ T cells

Another main function of Tregs is modulating CD8+ effector T cells. These cells play a critical role by producing antiviral cytokines and removing virus-infected cells.^124^ CD8+ T cells can also produce inflammatory cytokines such as TNF, which then activate microglia and other immune cells.^125^ Recent studies have found an increased number of CD8+ T cells present in patients with Alzheimer’s disease, which result in an increase in neuroinflammation.^126,127^ Although, other studies found no significant difference in CD8+ T cell frequencies compared with healthy controls.^59^ These inconsistencies may exist due to differences in the stage of disease studied. Despite this, it has been shown that Tregs can modulate CD8+ T cell expansion and differentiation^128,129^ to prevent further neuroinflammation. Although, the mechanisms behind this are not well understood. One study showed that IL-2 produced by CD8+ T cells expand Tregs, which then limit CD8+ cell responses.^129^ Thus, increasing Tregs can modulate CD8+ T cells, and in turn, can modulate neuroinflammation through this pathway. Exploring CD8+ T cells further could reveal a deeper insight into Alzheimer’s disease pathology, as their mechanisms in Alzheimer’s disease remain largely unknown.

Terminally differentiated effector T cells (TEMRA) are associated with CD and Alzheimer’s disease, having a more cytotoxic phenotype, also connected with neurodegeneration, neuroinflammation and plasma markers of AD.^130^ More specifically, CD8+ TEMRA cells were shown to be associated with neural injury and Alzheimer’s disease, whereas CD4+ TEMRA have a role in the interplay of inflammatory mechanisms and CD8+ activation.^130^

### Tregs reduce amyloid burden

There is a great focus within the research placed on Aβ plagues in Alzheimer’s disease as they are established as one of the most significant hallmarks of Alzheimer’s disease. Investigating ways of reducing Aβ plagues is a continuing approach in Alzheimer’s disease drug development, and many studies have looked at how Tregs effect Aβ plagues. Some studies have shown that a depletion of Tregs results in increased Aβ plagues.^59,131^ Supporting this, other studies have demonstrated that increased levels of Tregs lead to increased Aβ plague clearance.^39,61‐63,132‐163^ Contrasting this, Dansokho *et al*.^60^ found that there was no significant difference in Aβ burden in Treg-depleted APP/PS1 and non-depleted mice, although it was found that decreases in plaque-associated microglial activity and it was concluded that Tregs have a neuroprotective effect on Alzheimer’s disease models. The reasons behind the inconsistent finds are unclear, although it may be due to the modulation of Tregs at different stages of disease. Dansokho *et al*. altered Treg levels when mice were 5–6 weeks of age, compared to Treg modulation in 4- to 5-month-old mice in a conflicting study by Baruch *et al.*^133^ Despite findings by Dansokho *et al*., most researchers have come to the consensus that increasing levels of Tregs does reduce or prevent the development of Aβ plagues. Taken together, the findings still indicate that increasing levels of Tregs in Alzheimer’s disease is beneficial in reducing/preventing symptoms and therefore must be explored further to potentially develop a new drug type of Alzheimer’s disease.

### Tregs reduce phosphorylated tau

The second key hallmark in Alzheimer’s disease pathology is the NFT of phosphorylated tau protein. Whilst the impact increasing Tregs have on tau pathology is less prominent in the literature compared to the effect on Aβ plagues, it is necessary to discuss tau due to its prominence in the disease pathology. Data from several studies suggest that ongoing inflammation from microglia in Alzheimer’s disease facilitates tau phosphorylation,^73,134^ therefore implying that increasing Treg levels should prevent/reduce phosphorylated tau NFT, because Tregs reduce microglia and inflammation. A study by Yang *et al*.^62^ demonstrated mitigation of tau deposits by *ex vivo*-expanded Tregs, suggesting that Tregs can play a role in reducing tau Alzheimer’s disease pathologies. Another study found that Alzheimer’s disease patients’ levels of natural Tregs are correlated with the levels of NFT and Tau.^53^ Levels of tau have also been shown to predict the rate of CD.^135,136^ As increasing Tregs can restore cognitive function,^59,60^ this together implies that increasing Tregs could also reduce levels of tau. Adding to this, it has been shown that Tregs suppress microglia, which have been shown to accelerate development of NFT.^63^ It would be interesting to see more studies focusing on the impact of increased Tregs on tau pathogenesis in Alzheimer’s disease as there is limited research available on this concept, although it is clear from current research that there are various mechanisms triggered by an increase in Tregs that lead to reduced tau pathogenesis.

### Chimeric antigen receptor (CAR) Tregs immunotherapy for Alzheimer’s disease

There have been some significant advances in cancer therapy using CAR T cell therapy and this concept, using engineering CAR-Treg cells for treating patients with Alzheimer’s disease as an immunotherapy, despite there being numerous challenges to this approach.^137,138^ Designing an antigen target that is appropriate for the required immune outcome being one of the issues to address. Another being maintaining Treg phenotype and stability of function over time. Pathogenic conversion was observed for Tregs to TH17 cells in autoimmune arthritis.^139^

### Limitations of Treg infusions

#### Tregs act as an immunosuppressant

Although Tregs have an important immunoregulatory role, some immunosuppressant role of Tregs in Alzheimer’s disease is now recognized within the literature^42,54,133^; however, there is a relatively small portion of the literature that is concerned with the control of the immunosuppressive function of Treg infusions. To date, several studies have explored the relationship between immunosuppressants and cancer, as well as the importance of immune system equilibrium.^45,140,141^ The body needs an effective immune response to fight infections and keep cancer at bay^142^; however, if this immune response is overactive, then immune cells can cause tissue damage. This is known as autoimmune disease.^140,141,143^ Emerging theories of Alzheimer’s disease propose that Alzheimer’s disease is an autoimmune disease,^144^ which is supported by many of the mechanisms of Tregs used to reduce Alzheimer’s disease pathologies.^41,42,59^

One issue of using immunosuppressants is that they can weaken the whole body’s immune system and leave patients vulnerable to infections.^145^ This concern is highlighted by Ochs *et al*., who showed that decreasing Treg levels can assist with immunity against pathogenic microorganisms,^146^ suggesting increasing Treg levels may decrease immunity against infections. Supporting this, one study using a viral model for multiple sclerosis reported higher Treg frequencies to suppress immune response and increased viral replication; however, it was also found that Tregs did reduce immunopathology by reducing neuroinflammation.^147^ Furthermore, a 2021 study has suggested a link between mortality and the routine use of immunosuppressants in SARS-CoV-2 (COVID-19) patients.^148^ However, Harden *et al*.^149^ studied the use of Treg therapy in kidney transplants and concluded that Treg therapy was safe and resulted in fewer incidences of opportunistic infection in comparison to patients who were treated with other immunosuppressants. Despite this suggesting Treg infusions are safe, it is important to note that Harden *et al*. only show that Treg infusion are safer than other immunosuppressants that are typically associated with high risks.^145^ Whilst there are currently no data available on these risks in Alzheimer’s disease, most studies on other diseases appear to be consistent in findings that increased levels of Tregs result in increased risk of infection,^146,147,149^ suggesting that more research and consideration of risks is vital to produce a safe treatment.

As people age, their immune system naturally weakens.^150^ This is an important consideration relating to Alzheimer’s disease treatment as the prevalence of Alzheimer’s disease also increases with age^14‐16^; thus, many of the older patients may not be suitable candidates for immunosuppressant Treg infusions and concerns with safety of potential drugs arise. Studies have demonstrated that immunosuppressants can have dangerous consequences in older patients who may have compromised immune systems.^151^ However, more recent studies on the use of immunosuppressants to treat Crohn’s disease found no difference in the efficacy and safety of immunosuppressants between older and younger patients.^152^ Discrepancies in the data imply that the use of Tregs in older patients with Alzheimer’s disease may need to be assessed on an individual basis to assess the condition of their immune system to determine whether patients are at high risk of infection and side effects.

### Cancer and autoimmunity

**Figure 3:**
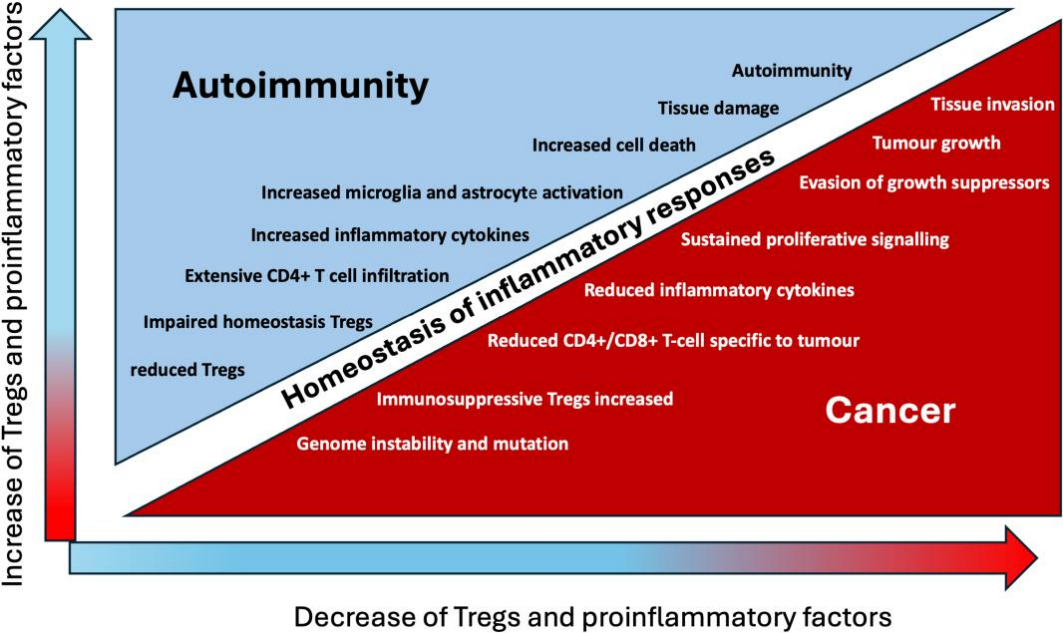
**Balancing autoimmunity and cancer.** Represent the fine balance of regulating the immune response and maintaining homeostasis in tissues. In immunotherapies, excessive suppression of the immune system can result in an increased risk of cancer,^153^ whereas an excessive enhancement of the immune system can result in autoimmune diseases.^145^ Image adapted from Desai *et al*.^145^ Singh *et al*.^152^, Curiel *et al*.^153^ and ^154^.

One of the biggest concerns with *ex vivo* expanded Treg infusions is the potential increased risk for cancer. Due to the immunosuppressive role of Tregs, theoretical speculations about the relationship between Tregs and cancer arise (Fig. 3). Whilst the literature on increasing Treg levels as a therapeutic for Alzheimer’s disease fails to mention this potential problem, other studies have found an increased frequency of Tregs in peripheral blood of cancer patients compared with healthy controls. These studies also show that Tregs accumulate within tumours, implying there is a clear link between Treg levels and cancer.^155,156^ One study by Curiel *et al*.^153^ describes increased tumour Tregs to predict reduced survival and treatment response, suggesting that Tregs may a significant role in cancer development. Conversely, another study showed that Treg levels present in renal cancer tumours did not predict survival,^157^ with a third study finding that an increased number of Tregs predicted improved survival in Hodgkin lymphoma patients.^158,159^ The inconsistencies in the data from these studies may be due to the differences in cancer types or the methodology used in these studies used to identify Tregs using *Foxp3* expression.^153‐158^ Not all Foxp3-expressing cells are functional Tregs.^160^ Thus, without a functional test, inaccuracies in identifying Tregs may occur. However, it remains necessary for research to investigate the relationship between Tregs and cancer further before moving forward to clinical trials using Tregs to treat Alzheimer’s disease (Figure 3).

Furthermore, similarly to AD,^14‐16^ cancer is often considered to be an age-related disease.^161^ Meaning that patients with Alzheimer’s disease are already at a higher risk of developing cancer than the average population,^162^ and Tregs may also increase this risk further. It is, therefore, recommended that any clinical trials involving increasing Tregs in patients with Alzheimer’s disease should carefully consider this potential risk when selecting participants. This major limitation in the use of Treg therapies is largely ignored by animal model studies, although the reason for this remains unclear. This may be due to the use of antigen-specific technologies, although there is still a risk of non-specific immunosuppression with the use of polyclonal Tregs.^39,163^

### Lack of understanding of mechanism of suppression, survival and plasticity

Due to the potential risks associated with overexpression of Tregs, there is a clear need to understand the mechanism with Tregs and their interactions with other cell types. The high need for a balance within multiple aspects of the treatments means that an understanding of how these cells can survive or be suppressed is essential to produce a safe and effective drug. For example, the immunosuppressant mechanisms can lead to an increased risk of cancer or reduced ability to fight infections.^155,156^ Tregs have been shown to exhibit high levels of plasticity due to interactions with cytokines. For example, a subset of Tregs has been shown to lose expression of *Foxp2* in a depletion of IL-2 and pro-inflammatory cytokines. This results in a change in phenotype to an effector T cell.^164,165^ Instability of the Foxp3 protein is a concern for researchers exploring Treg-based therapies, as the differentiation of Tregs into effector T cells (eTregs) could lead to increased inflammation.^125^ IL-2 could potentially be used to prevent plasticity of Tregs, due to the role played in Tregs survival and stability.^110,111^ IL-2 has been used in combination with Treg therapies to increase the longevity of Treg survival,^60,166^ demonstrating the potential to target Treg control using IL-2. Despite this, the mechanisms of suppression, survival and plasticity of Tregs remain partially unknown. Research focused on better understanding the mechanisms behind Treg cell stability are needed to control and retain stable Tregs to produce a predictable, safe and effective treatment for Alzheimer’s disease.

### Limited long-term effects

Long-term effects are an important factor to consider when assessing the safety of any new treatment. Due to the novelty of Treg therapies, there are limited data available on the long-term impact of these therapies. A Treg adoptive cell therapy trial in renal transplant patients, where patients received *ex vivo* expanded polyclonal Tregs, reported no record of adverse effects, infections, or rejections 2 years post-transplant.^163^ The data from this study provide enough information to support the safety of Treg therapy in Alzheimer’s disease clinical trials, although longitudinal studies over longer periods of time would confirm this. In 2018, Thonhoff *et al*.^167^ published a study on Treg infusions in 3 ALS patients and found that infusions slowed progression rates of disease, again suggesting that Treg infusions are safe and effective in treating neuroinflammation. Although Alzheimer’s disease and ALS are vastly different in many ways, both conditions are characterized by neuroinflammation and activation of microglia. Thus, Thonhoff *et al*.^167^ provide promising results for the therapeutic potential of Treg infusions in AD. Clinical trials for Treg therapies are yet to commence; however, the data from studies in other diseases show that treatment should be safe and effective in Alzheimer’s disease.

Given the role of oxidative stress, it is apparent that Tregs that differentiate after the infusion treatments would continue to have the same issues of reduces frequencies and function. Few trials have addressed the long-term survival of Tregs from infusions, due to the increased apoptosis due to low IL-2 levels,^168^ and they may not be able to survive as intended. As previously mentioned, clinical trials have established that Treg populations can be restored and support the suppressor function of Tregs.^64^ Treatment using *ex vivo* expanded Tregs may require continuous treatment for the desired effects, although it is unclear how frequently infusions would be necessary to maintain Treg levels. However, mice model trials^60^ and a clinical trial exploring a combined therapy of Tregs, with a dose of IL-2 has been recently published, demonstrating the beneficial effects of IL-2 on Treg functions.^166^ It is unclear whether further doses of IL-2 were required, although this demonstrates a potential resolution to this issue that needs to be investigated further.

A 2017 study described being unable to explore the functions of Tregs due to an insufficient number of cells from participants.^58^ This highlights another issue with this type of treatment. Many cells are required for polyclonal expansion and if doctors cannot extract enough cells to expand, patients may not be suitable for treatment using *ex vivo* expansion techniques,^163^ although adoptive transfer may still be feasible.

### Cost of treatment

When investigating a potential new treatment for a debilitating disease such as Alzheimer’s disease, therapies using *ex vivo* expanded cell technologies are notoriously expensive. Finding ways to reduce these costs and make treatments assessable is crucial to facilitate the widespread usage and success of new technologies. In the UK, the National Institute for Health and Clinical Excellence (NICE) uses an Incremental Cost Effectiveness Ratio (ICER) of up to £30 000 per quality-adjusted life year (QALY) as the system to approve new drug therapies.^169^ Given that a similar Treg therapy for cancer (Carvykti) reportedly costs $465 000 for a single infusion^87^ and that there is evidence discussed above to suggest that Treg therapies in Alzheimer’s disease would require multiple infusions, the likelihood of such therapies being approved in the UK appears next to impossible. This highlights the importance of exploring new techniques and methods to discover assessable drug therapies for all. Whilst Tregs can prevent Alzheimer’s disease symptoms and pathologies, the search for a cost-effective alternative to Treg infusions is vital in the treatment of Alzheimer’s disease globally. It should be mentioned that *in vivo* Treg expansion therapy using IL-2 immunotherapy is a cheaper and viable option compared to *ex vivo* Treg expansion therapy.^67^

### Conclusion and future research directions

Within the past decade, there has been a shift in focus in Alzheimer’s disease research towards immunotherapy and the therapeutic potential of Treg-based therapies and researchers remain cautiously optimistic about the potential of these immunotherapy approaches. Given the current literature, it can be concluded that increasing levels of Tregs can prevent neuroinflammation and the hallmark symptoms and pathologies of Alzheimer’s disease; however, the plethora of interactions involving Tregs are not fully understood. Promising results have demonstrated the ability of Tregs to reduce neuroinflammation in Alzheimer’s disease models. This study is unable to encompass the entire web of cellular pathways in which Tregs play a role in, however, a few significant pathways mentioned in the literature have been explored. Treg therapies act to modulate key immune components of the CNS, such as microglia, astrocytes and cytokines, to reduce Aβ and tau pathologies in animal models. Furthermore, Treg infusions have been demonstrated to slow CD and the progression of memory loss in Alzheimer’s disease mice models, highlighting the undeniable potential of the use of Treg therapies for Alzheimer’s disease. However, the inconsistencies of findings between studies demonstrate a lack of understanding of these mechanisms.

Tregs can be modulated by various factors. This review focuses on oxidative stress as an example. Increased oxidative stress in Alzheimer’s disease is reported to result in a depletion of Tregs. However, it is difficult to determine whether this is a cause or symptom of the disease. A deeper understanding how Tregs are modulated within the CNS may provide insight into the onset of Alzheimer’s disease and new prevention techniques. It would be interesting to see further research investigating this relationship further, including the effects using Treg therapies alongside an antioxidant-rich diet and IL-2. Tregs are also modulated by many cytokines, such as IL-6 and IL-2, understanding the complex interactions of these may allow for therapies that naturally increase Treg levels without the need for infusions.

The successful use of Treg therapies in organ transplants, ALS and Crohn’s disease demonstrates the safety and efficacy of these novel treatments; however, there are limited data on the long-term consequences. Whilst the potential is promising, further research into understanding the complex mechanisms surrounding Tregs is crucial to fully understand the risks of increasing frequencies of Tregs in patients with Alzheimer’s disease. For example, without the knowledge of how these infused cells survive, migrate and proliferate, questions around the dangers of non-specific immunosuppression, autoimmunity and cancer remain. A greater understanding of Treg homeostasis and ‘normal’ function needs to be understood by comparing control groups with patients with Alzheimer’s disease to ascertain safe levels for new clinical trials.

The effects of ROS, OS and Treg function must be investigated in patients with neurogenerative disorders to ascertain if combination therapies could reduce OS while enhancing Treg function. Indeed, can the immunotherapy infuse functional Tregs or could immunotherapy enhance the environment to activate existing Tregs and the T-cell differentiation? Trials to explore further the impact of *in vivo* Treg expansion using IL-2 are eagerly anticipated when translated to human patients with neurodegenerative conditions.^64^

Further, new generations of therapy with novel approaches of combination therapies, using different cocktails of monoclonal antibodies, cytokines and CAR Tregs may prove to be successful immunotherapy approaches for Alzheimer’s disease. CAR constructs are able to bind to neurotoxic Aβ monomers, deposited extracellularly.^138^

A recent proof of concept study showing that adoptive transfer of specific TCR Aβ regulatory Tregs restores brain homeostasis in mice may be an important new immunotherapy strategy in human AD patients.^63^

It is important to note that much of the research covered in this review explores animal models and human studies need to be trialled to confirm the findings from bench to bedside. Careful consideration must be taken when selecting participants for clinical trials of Treg-based therapies to reduce potential risks. Although until the extensive modulation and activation triggered by Tregs is understood, researchers may not truly comprehend the long-term side effects. A potential limitation of the current study is that the scope may be too broad to provide a comprehensive review of the impact of Tregs. However, Alzheimer’s disease progression is caused by a multitude of factors, and it appears that Treg therapies can target many of these, suggesting a bright future as a potential treatment for AD.

## Data Availability

Data sharing is not applicable to this article as no new data were created or analysed in this study.
